# Serum hydroxybutyrate dehydrogenase as an early predictive marker of the severity of acute pancreatitis: a retrospective study

**DOI:** 10.1186/s12876-020-01521-7

**Published:** 2020-11-20

**Authors:** Weiming Xiao, Weili Liu, Ling Yin, Yong Li, Guotao Lu, Xinnong Liu, Weijuan Gong, Yanbing Ding, Mei Wang, Zhigang Yan

**Affiliations:** 1 Pancreatic Center, Department of Gastroenterology, Affiliated Hospital of Yangzhou University, Yangzhou University, Yangzhou, Jiangsu China; 2 Institute of Gastroenterology, Affiliated Hospital of Yangzhou University, Yangzhou University, Yangzhou, Jiangsu China; 3 Department of Intensive Care Unit, Affiliated Hospital of Yangzhou University, Yangzhou University, Yangzhou, Jiangsu China; 4 Department of General Surgery, Affiliated Hospital of Yangzhou University, Yangzhou University, Yangzhou, Jiangsu China

**Keywords:** Acute pancreatitis, HBDH, LDH, Organ failure, SIRS

## Abstract

**Background:**

To investigate the value of serum hydroxybutyrate dehydrogenase (HBDH) level, an isozyme of lactate dehydrogenase, in evaluating the severity of acute pancreatitis (AP).

**Methods:**

Patients diagnosed with AP from January 2013 to December 2018 were included in this retrospective study. Patients were divided into the normal serum HBDH levels group (n-HBDH group) and the high serum HBDH levels group (h-HBDH group) according to the criteria HBDH ≥ 182 U/L after admission. The demographic parameters, laboratory data and the severity of AP in the two groups were compared. The receiver operating curve (ROC) was used to evaluate the efficacy of serum HBDH in predicting persistent organ failure and systemic inflammatory response syndrome (SIRS).

**Results:**

A total of 260 AP patients were enrolled, including 176 cases in the n-HBDH group and 84 cases in the h-HBDH group. The incidence of SIRS and organ failure in the h-HBDH group were significantly higher than those in n-HBDH group (both *P* < 0.001). In addition, the HBDH level was significantly decreased in 110 patients who were re-measured after AP treatment. The serum HBDH levels were positively correlated with Atlanta classification, Ranson score, and BISAP score (all *P* < 0.05). ROC analysis showed that a serum HBDH cut-off point of 195.0 U/L had optimal predictive value for the development of persistent organ failure (AUC = 0.778) and 166.5 U/L for the development of SIRS (AUC = 0.724).

**Conclusion:**

The elevated serum HBDH in early stage of AP is closely related to the adverse prognosis of AP patients, which can be used as a potential early biomarker for predicting the severity of AP.

## Background

Acute pancreatitis (AP) is one of the most common gastrointestinal diseases of hospitalised patients in China, the United States and other countries, and its incidence rate continues to rise globally. The incidence and mortality of AP were estimated to be 33.74 cases per 100,000 person-years and 1.60 deaths per 100,000 person-years, respectively [1]. About 80% of AP cases are self-limiting with no complications, and the remainder progresses to severe cases with local or systemic complications [2], systemic inflammatory response syndrome (SIRS) and organ failure, which ultimately leads to a significant increase in mortality [3].

Early assessment of the severity of AP is a key factor in determining treatment strategies [4]. At present, some clinical multi-factor scoring systems, including Ranson, BISAP, Glasgow coma and Acute Physiology and Chronic Health Evaluation II (APACHE II) scores are used to predict the occurrence of SAP [5, 6]. However, according to published reports, different scoring systems have a certain predictive function on the severity of AP, but all these methods are complicated and difficult to obtain the preliminary data [7, 8]. Therefore, there is an urgent need for a novel, simple and effective evaluation method, or indicator to predict the severity of AP.

Lactate dehydrogenase (LDH) is a key enzyme in anaerobic glycolysis, catalysing the mutual conversion of lactic acid and pyruvate [9]. The level of serum LDH is associated with sepsis, AP, and tumour growth and metastasis [10–12]. Levels of hydroxybutyrate dehydrogenase (HBDH) was measured by using α-ketoacid as a substrate. Since the H subunits of HBDH have a high affinity for α-ketoacid, HBDH mainly represents the activity of LDH1 and LDH2 which contains more H subunits. Isozymes LDH1 and LDH2 dominate in the myocardium, red blood cells, and kidneys, which show more affinity for α-ketoacid. Therefore, in recent years, scientists have gradually focused on the clinical application value of LDH isozyme HBDH. Studies have found that serum HBDH level significantly changes in myocardial infarction, atherothrombotic and liver injury [13–15].

At present, LDH has been incorporated into the Ranson scoring system to assess the severity of AP and predict persistent organ failure in AP [16]. Whether there are significant changes in circulating HBDH levels in AP and whether they have predictive value for AP has not been reported. The purpose of this study was to determine the serum levels of HBDH in AP and find a possible correlation between HBDH levels and severity of AP.

## Methods

### Inclusion and exclusion criteria

Patients diagnosed with AP from January 2013 to December 2018 in the Department of Gastroenterology, Yangzhou, China, were included in our study. Diagnosis of AP was based on the existence of two or more of the following criteria: (1) typical clinical symptoms with persistent abdominal pain; (2) serum amylase and/or lipase levels higher than three times the normal upper limit; (3) characteristic results of abdominal imaging [17]. Exclusion criteria included any of the following: (1) < 18 years of age; (2) acute pancreatitis in pregnancy; (3) traumatic pancreatitis; (4) pancreatic cancer; (5) have undergone AP treatment in other hospital or departments; (6) patients hospitalized 3 days (72 h) after AP onset; (7) patients without serum HBDH values.

### Data collection

Laboratory data was obtained from a blood screening test during hospitalisation. Total quantity of HBDH (BIOSINO) was tested by an enzymatic kit by automatic biochemical analyzer (measure absorbance at 340 nm), with a reference value ranged from 72 to 182 U/L. The basic principle is that HBDH catalyses the reduction of α-ketobutyric acid to α- hydroxybutyric acid and catalysed the oxidation of reduced nicotinamide adenine dinucleotide (NADH) to oxidized nicotinamide adenine dinucleotide (NAD+). In order to obtain relevant demographics, physiological variables and disease severity, the electronic medical records and laboratory test results of all patients were checked by an independent doctor. This study was conducted in accordance with the principles of the Helsinki Declaration. Due to the retrospective characteristics of the study from 2013 to 2018, informed consent was waived and the study was approved by the ethics committee of the affiliated hospital of Yangzhou University.

### Severity assessment of AP

According to the Atlanta classification revised in 2012 [17], AP was divided into three groups: mild acute pancreatitis (MAP), moderately severe acute pancreatitis (MSAP) and severe acute pancreatitis (SAP). MAP refers to AP patients with no organ failure, and no local or systemic complications. MSAP refers to those suffering from transient organ failure and/or local or systemic complications (within 48 h). Finally, patients with SAP are those who have organ failure and/or local or systemic complications which last longer than 48 h and local complications involved peripancreatic fluid collections, pancreatic and peripancreatic necrosis (sterile or infected), pseudocyst and walled-off necrosis (sterile or infected). Diagnosis of organ failure required assessment of the respiratory, circulatory, and renal functions. According to the Atlanta classification revised in 2012, persistent organ failure was defined by a score of 2 or more over a period of more than 48 h for one of these three organ systems based on the modified Marshall scoring system [17].

### Diagnostic criteria for systemic inflammatory response syndrome (SIRS)

SIRS was defined by the existence of two or more of the following four criteria: (1) temperature > 38 °C or < 36 °C; (2) heart rate > 90 beats/min or hypotension; (3) tachypnoea (> 20 breaths/min) or hyperventilation (PaCO2 < 32 mmHg); (4) Peripheral blood leukocyte count > 12 × 10^9^/L or neutral rod-shaped granulocyte ratio > 10%. In addition, other conditions that may cause the above acute abnormal changes should be excluded [17].

### Statistical analysis

All continuous variables were represented as the mean ± standard deviation (SD). Data were analysed with SPSS 16.0 (SPSS Inc., Chicago, USA). Independent sample t test and chi-square test were used to compare continuous variables and categorical variables, respectively. Pearson correlation analysis was used to examine the correlation between serum HBDH levels and other laboratory indicators. Receiver operating characteristic (ROCs) curves were applied to assess the sensitivity and specificity of the indicators by GraphPad Prism 5.0 software. A bilateral *P* < 0.05 was considered to be a statistically significant difference.

## Results

### Comparison of clinical characteristics

A total of 842 patients diagnosed with AP from 2013 to 2018 were reviewed in this study, 582 patients were excluded according to the exclusion criteria, and finally 260 AP patients were enrolled. Of them, 176 (67.7%) patients had normal serum HBDH levels, whereas 84 patients (32.3%) had elevated serum HBDH levels.

Of all 260 patients, 162 patients (62.3%) were male, and the average age was 51.7 years old. Hypertriglyceridemia was the most common AP etiology (40.4%), followed by biliary diseases (24.2%) and alcohol consumption (14.2%), and other causes accounted for 21.2% of cases. Among these patients, 81 (31.2%) patients suffered from organ failure, and 75 (28.8%) had SIRS, as showed in Table 1.

**Table 1:** Comparison of clinical characteristics and outcomes between AP patients with versus without high serum HBDH levels

	ALL	High HBDH	Normal HBDH	*P* value
	*N* = 260	*N* = 84	*N* = 176	
Age (mean ± SD), years	51.7 ± 16.1	52.4 ± 16.6	51.3 ± 16.0	0.617
Male sex, N (%)	162 (62.3)	48 (57.1)	114 (64.8)	0.235
Smoking, N (%)	80 (30.8)	26 (31.0)	54(30.7)	0.965
Drinking, N (%)	57 (21.9)	22 (26.2)	35 (19.9)	0.251
Underlying diseases, N (%)				
Diabetes	52 (20.0)	19 (22.6)	33 (18.8)	0.466
Hypertriglyceridemia	65 (25.0)	21 (25.0)	44 (25.0)	1.000
hypertension	93 (35.8)	36 (42.9)	57 (32.4)	0.100
NAFLD	108 (41.5)	32 (38.1)	76 (43.2)	0.436
Etiology, N (%)				0.241
Biliary	63 (24.2)	22 (26.2)	41 (23.3)	
Alcohol	37 (14.2)	11 (13.1)	26 (14.8)	
Hypertriglyceridemia	105 (40.4)	39 (46.4)	66 (37.5)	
Others	55 (21.2)	12 (14.3)	43 (24.4)	
Severity, N (%)				< 0.001***
MAP	179 (68.8)	40 (47.6)	139 (79.0)	
MSAP	65 (25.0)	32 (38.1)	33 (18.8)	
SAP	16 (6.2)	12 (14.3)	4 (2.3)	
OF, N (%)	81 (31.2)	44 (52.4)	37 (21.0)	< 0.001***
SIRS, N (%)	75 (28.8)	39 (46.4)	36 (20.5)	< 0.001***
Mortality, N (%)	3 (1.2)	2 (2.4)	1 (0.6)	0.201

Data are presented as the means ± standard deviation or interquartile range.*P < 0.05, **P < 0.01, ***P < 0.001. *P* values were determined by Student’s *t*-test for continuous variables and the chi-square test for categorical variables

### Serum HBDH levels and severity of AP

There was no significant difference in terms of age, gender, history of tobacco and alcohol consumption, and underling disease between the n-HBDH and h-HBDH groups. Compared with the n-HBDH group, the incidence of MAP was lower (47.6% vs. 79.0%), whereas the incidence of SAP (14.3% vs. 2.3%) and MSAP (38.1% vs. 18.8%) were higher in the h-HBDH group (both *P* < 0.001). Moreover, there was a higher proportion of organ failure (52.4% vs. 21.0%) and SIRS (46.4% vs. 20.5%) in the h-HBDH group, as shown in Table 1 (both *P* < 0.001). In addition, in the h-HBDH group, patients had higher levels of white blood cell (WBC), percentage of neutrophils (N%), LDH, direct bilirubin (DB), aspartate transaminase (AST), alanine transaminase (ALT), gamma glutamyl transferase (GGT), blood urea nitrogen (BUN), C-reactive protein (CRP) and calcium ion (Ca^2+^) (all *P* < 0.05). Whereas, there was no significant difference in the serum levels of creatinine (Cr) between the two groups, as shown in Table 2. A total of 3 patients died, including 2 patients with elevated HBDH and 1 patient with normal HBDH, there was no significant difference in mortality between the n-HBDH and h-HBDH groups.

**Table 2:** Comparison of laboratory data between AP patients with versus without high serum HBDH levels

	ALL	High HBDH	Normal HBDH	*P* value
	*N* = 260	*N* = 84	*N* = 176	
WBC, × 10*9/L	12.4 ± 5.1	13.9 ± 5.6	11.7 ± 4.7	0.002**
N%	79.7 ± 13.5	83.0 ± 9.8	78.1 ± 14.8	0.007**
LDH,U/L	253.1 ± 131.1	335.9 ± 160.1	213.5 ± 91.7	< 0.001***
DB, μmol/L	6.3 ± 14.1	9.0 ± 19.8	5.0 ± 10.2	0.080*
AST, U/L	91.2 ± 145.9	130.6 ± 202.9	72.4 ± 104.3	0.015*
ALT, U/L	88.7 ± 133.7	121.2 ± 186.6	73.2 ± 95.8	0.028*
GGT, U/L	183.2 ± 273.9	269.5 ± 360.7	142.0 ± 209.9	0.003**
BUN, mmol/L	5.1 ± 2.2	5.5 ± 2.8	4.9 ± 1.9	0.046*
CRP, mg/L	33.7 ± 36.8	42.3 ± 45.1	29.6 ± 31.4	0.022*
Ca^2+^, mmol/L	2.3 ± 0.2	2.2 ± 0.2	2.3 ± 0.2	< 0.001***
Cr, μmol/L	63.5 ± 25.1	67.0 ± 33.2	61.8 ± 20.1	0.189
Glu, mmol/L	8.8 ± 0.3	9.6 ± 0.6	8.4 ± 0.4	0.066

Data are presented as the means ± standard deviation or interquartile range.*P < 0.05, **P < 0.01, ***P < 0.001. *P* values were determined by Student’s *t*-test for continuous variables and the chi-square test for categorical variables

### Serum HBDH levels and clinical scoring systems in AP

**Fig. 1:**
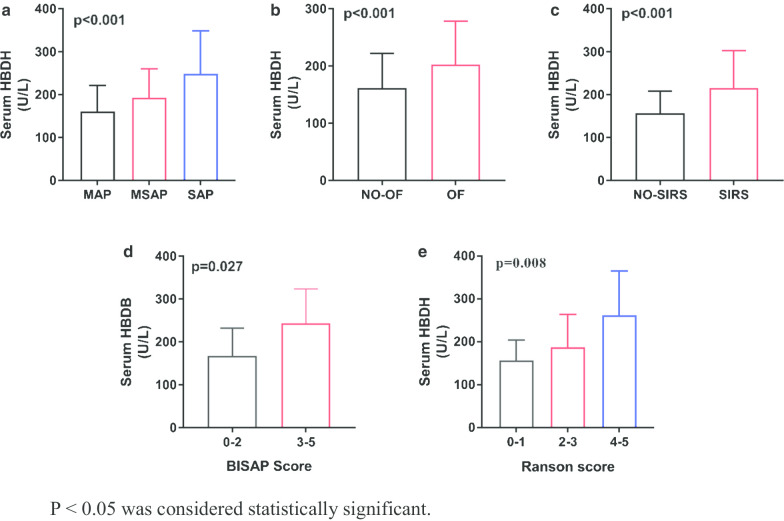
Comparison of serum HBDH concentrations by Atlanta classification, Ranson, BISAP score. *P* < 0.05 was considered statistically significant

**Fig. 2:**
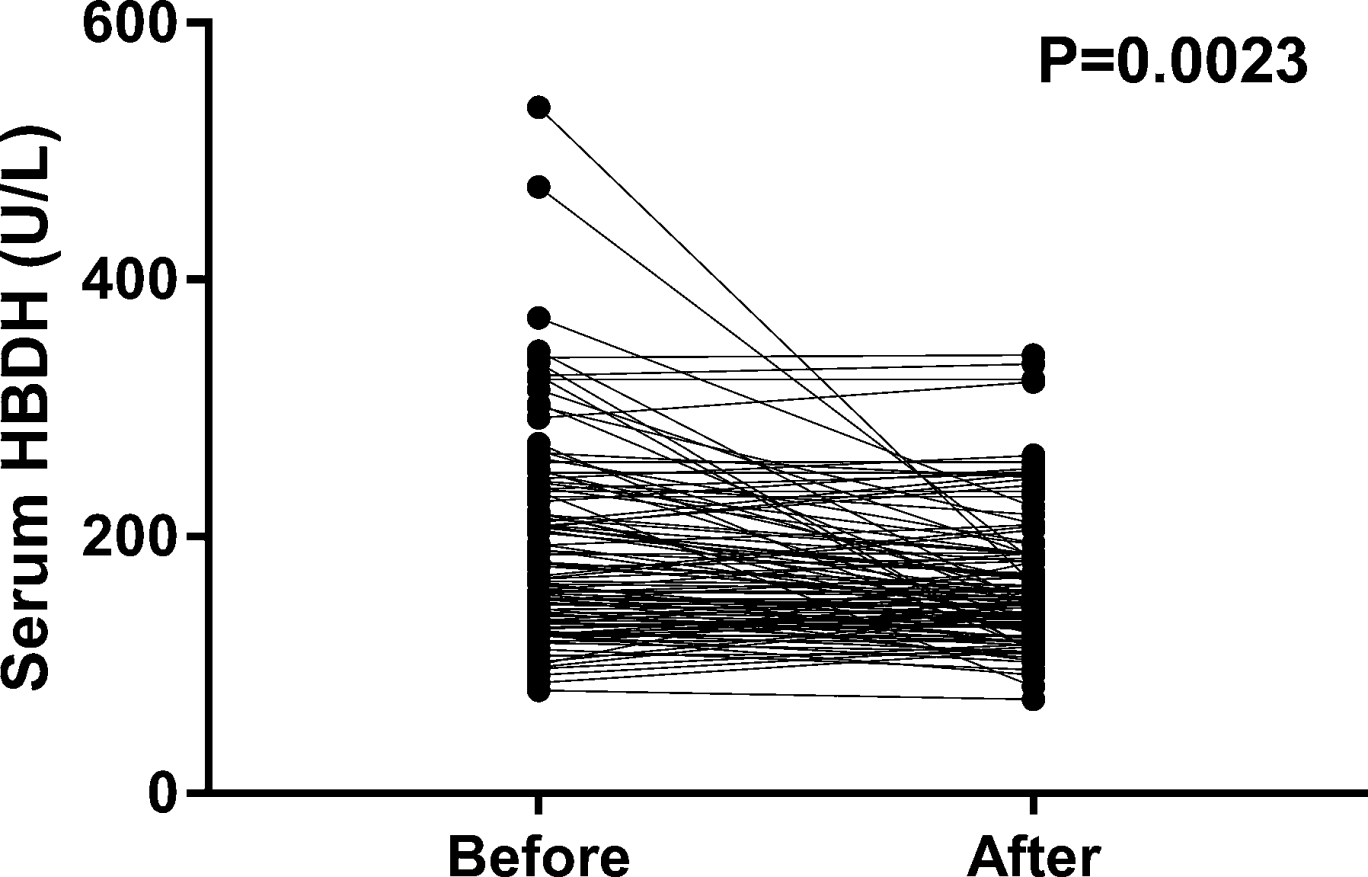
Dynamic changes of HBDH in AP patients

The scoring systems for the severity of AP are varied and each has its own merits and demerits. We observed correlation between serum HBDH levels and clinical scoring systems. As shown in Fig. 1, serum HBDH levels were significantly related to the clinical scoring systems of AP. Serum HBDH levels were significantly increased in patients with organ failure and SIRS, and were positively correlated with Atlanta classification, Ranson score, and BISAP score. Of the 260 AP patients with elevated HBDH levels, 110 patients had their HBDH levels re-measured during hospitalisation, which significantly decreased from 191.5 ± 7.459 U/L to 163.6 ± 5.053 U/L (Fig. 2).

### Correlation between serum HBDH levels and other clinical indicators

To further evaluate the predictive value of HBDH level for AP prognosis, correlation analysis was carried out between the serum HBDH level and other clinical markers (all these data are from the same time). As shown in Table 3, serum HBDH levels were positively correlated with the serum levels of WBC (R = 0.273; *P* < 0.001), N% (R = 0.162; *P* = 0.009), LDH (R = 0.467; *P* < 0.001), AST (R = 0.152; *P* = 0.014), GGT (R = 0.150; *P* = 0.015), BUN (R = 0.165; *P* = 0.008), triglyceride (TG, R = 0.195; *P* = 0.002), cholesterol (CHO, R = 0.132; *P* = 0.037), glucose (GLU, R = 0.171; *P* = 0.006), CRP (R = 0.182; *P* = 0.003). Whereas, there was no significant correlation between serum HBDH and Ca^2+^, ALT, CRP, DB, high-density lipoprotein (HDL), and low-density lipoprotein (LDL).

**Table 3:** Correlation between HBDH and other clinical indicators in AP patients

Index	R (with HBDH)	*P* value	Index	R (with HBDH)	*P* value
WBC	0.273	< 0.001***	Glu	0.171	0.006**
N%	0.162	0.009**	CRP	0.182	0.003**
LDH	0.467	< 0.001***	DB	0.052	0.399
AST	0.152	0.014*	Ca2+	0.043	0.491
GGT	0.150	0.015*	Cr	0.107	0.084
BUN	0.165	0.008**	HDL	0.057	0.368
TG	0.195	0.002**	LDL	− 0.062	0.325
CHO	0.132	0.037*	ALT	0.103	0.096

*P < 0.05; **P < 0.01; ***P < 0.001

### ROC curve analysis of HBDH to diagnose organ failure

**Fig. 3:**
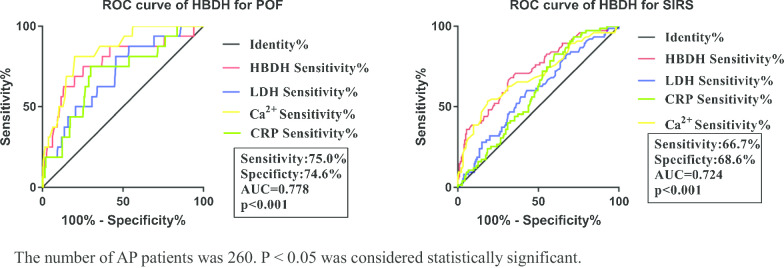
The ROC curve for determining the HBDH cut-off value for identifying POF and SIRS. The number of AP patients was 260. *P* < 0.05 was considered statistically significant

ROC curve analysis was performed to determine the cut-off value of HBDH for predicting AP with persistent organ failure or SIRS. As shown in Fig. 3, the results revealed that the area under the ROC curve of HBDH for persistent organ failure was 0.778 and the optimal cut-off level was 195.0 U/L, which provided a 75.0% sensitivity and a 74.6% specificity. The area under the ROC curve of HBDH for SIRS was 0.724 and the optimal clinical cut-off level was 166.5 U/L, which provided a 66.7% sensitivity and a 68.4% specificity. In addition, our study found that the ability of serum HBDH for predicting persistent organ failure or SIRS is superior to LDH and CRP.

## Discussion

AP is a non-infectious inflammatory disease that usually presents as self-limiting. Most AP cases do not require treatment interventions, but some of them suffer organ failure when the inflammatory response is excessive and can lead to a poor prognosis [18]. The duration of organ failure is the main criterion for the Atlanta classification revised in 2012. Some studies have found that the proportion of organ failure developed by AP in the Han population is between 8 and 20%, and even up to 40% in certain areas [19]. Our results showed that the incidence of organ failure caused by AP is about 31.3%, which is consistent with the above results. Prognosis of AP can be effectively improved if organ failure and SIRS are detected as early as possible and intervention measures are taken. In recent years, many scales, such as the CTSI, Ranson, BISAP, Glasgow coma and APACHE II scores, have been designed to predict the prognosis of AP. However, each scale has different limitations in clinical application due to its complexity, such as a higher number of indices and longer time span. So, it is necessary to find a clinically applicable biomarker to predict SAP.

Some studies have pointed out the value of LDH in AP disease assessment. Yin et al. found that the patient’s LDH level can be used as an indicator to assess the severity and prognosis of AP [20]. Cui et al. observed that serum LDH at admission was independently associated with POF in AP and hence may be a potential prognostic factor [11]. Komolafe et al. suggested that LDH can be used as an index for distinguishing between edema pancreatitis and necrotising pancreatitis [21].

Our study focused on the predictive value of serum HBDH in the early assessment of AP. HBDH is an isoenzyme of LDH exhibiting LDH activity. Previous studies have shown that HBDH can be used to assess the severity of systemic lupus erythematosus-associated liver injury diseases, intrahepatic cholestasis of pregnancy and AIDS-related pneumocystis pneumonia [22–24]. All these studies indicate the importance of serum HBDH as a clinical biomarker. In this study, 32.8% of patients had high serum HBDH levels. The incidence of SIRS and organ failure in the h-HBDH group was significantly higher than that in the n-HBDH group. In addition, the serum HBDH levels in patients significantly increased with organ failure and SIRS but significantly decreased after AP treatment. Serum HBDH levels were positively correlated with Atlanta classification, Ranson score and BISAP score. These results suggest that elevated serum HBDH levels are closely associated with the severity and poor prognosis of AP, but not with the pathogeny, gender, and age. Furthermore, based on the ROC curve analysis, we observed that the ability of HBDH in predicting persistent organ failure in AP is about 77.8%, which is significantly better than LDH and CRP. In addition, the ability of HBDH for predicting SIRS in AP is about 72.4%, which is the best among the four markers, and so serum HBDH may be more suitable for SAP prediction.

All kinds of inflammatory diseases involve cell injury. Cells injury or necrosis caused by different pathogens releases structurally different LDH isoenzymes, which can regulate the metabolism of lactate and pyruvate, resulting in increased lactate and reduced pyruvate production, and eventually aggravating the inflammation [25–28]. Ferriero et al. proved that galloflavin as an LDH inhibitor reduced hepatocyte necrosis and apoptosis in a mice model of hepatic failure [29]. Fantin et al. observed that LDH inhibitor can inhibit tumour growth and migration by reducing lactate release [30]. AP is a complex inflammatory syndrome that can develop into systemic inflammation and multiple organ failure. The protective role of LDH inhibitors in AP needs further exploration.

Our study has some limitations worth noting. Firstly, this is a retrospective study that only demonstrated the correlation between HBDH and AP, but could not clarify the specific role of HBDH in the development of AP. Secondly, the study was conducted in a single centre with a relatively small sample size and therefore, future large-scale cohort studies are warranted to confirm the predictive role of HBDH in AP.

## Conclusion

In conclusion, our results provide evidence that elevated serum HBDH indicates adverse prognosis in AP patients. HBDH can be used as an early marker to distinguish SIRS from organ failure clinically.

## Data Availability

The datasets analyzed during the current study are available from the corresponding author on reasonable request.

## Abbreviations

HBDH: α-Hydroxybutyrate dehydrogenase
NAFLD: Nonalcoholic fatty liver disease
MAP: Mild acute pancreatitis
MSAP: Moderately severe acute pancreatitis
SAP: Severe acute pancreatitis
BISAP: Bedside index for severity in acute pancreatitis
NADH: Reduced nicotinamide adenine dinucleotide
NAD +: Oxidized nicotinamide adenine dinucleotide
OF: Organ failure
POF: Persistent organ failure
SIRS: Systemic inflammatory response syndrome
AUC: Area under the curve
WBC: White blood cell
N%: Percentage of neutrophils
LDH: Lactate dehydrogenase
DB: Direct bilirubin
AST: Aspartate transaminase
ALT: Alanine transaminase
GGT: Gamma glutamyl transferase
BUN: Blood urea nitrogen
CRP: C-reactive protein
Cr: Creatinine
CHO: Cholesterol
HDL: High-density lipoprotein
LDL: Low-density lipoprotein
Glu: Glucose

## References

[1] Xiao AY, Tan MLY, Wu LM (2016). Global incidence and mortality of pancreatic diseases: a systematic review, meta-analysis, and meta-regression of population-based cohort studies. Lancet Gastroenterol Hepatol.

[2] Lankisch PG, Apte M, Banks PA (2015). Acute pancreatitis. Lancet.

[3] Hines OJ, Pandol SJ (2019). Management of severe acute pancreatitis. BMJ.

[4] Wu BU, Johannes RS, Sun X (2008). The early prediction of mortality in acute pancreatitis: a large population-based study. Gut.

[5] Di MY, Liu H, Yang ZY (2016). Prediction models of mortality in acute pancreatitis in adults: a systematic review. Ann Intern Med.

[6] Cho JH, Kim TN, Chung HH (2015). Comparison of scoring systems in predicting the severity of acute pancreatitis. World J Gastroenterol.

[7] Papachristou GI, Muddana V, Yadav D (2010). Comparison of BISAP, Ranson's, APACHE-II, and CTSI scores in predicting organ failure, complications, and mortality in acute pancreatitis. Am J Gastroenterol.

[8] Galen RS, Reiffel JA, Gambino R (1975). Diagnosis of acute myocardial infarction. Relative efficiency of serum enzyme and isoenzyme measurements. JAMA.

[9] Jovanovic P, Zoric L, Stefanovic I (2010). Lactate dehydrogenase and oxidative stress activity in primary open-angle glaucoma aqueous humour. Bosn J Basic Med Sci.

[10] Lu J, Wei ZH, Jiang H (2018). Lactate dehydrogenase is associated with 28-day mortality in patients with sepsis: a retrospective observational study. J Surg Res.

[11] Cui J, Xiong JX, Zhang YS (2017). Serum lactate dehydrogenase is predictive of persistent organ failure in acute pancreatitis. J Crit Care.

[12] Manerba M, Di IL, Govoni M (2017). Lactate dehydrogenase inhibitors can reverse inflammation induced changes in colon cancer cells. Eur J Pharm Sci.

[13] Dissmann R, Linderer T, Schröder R (1998). Estimation of enzymatic infarct size: direct comparison of the marker enzymes creatine kinase and alpha-hydroxybutyrate dehydrogenase. Am Heart J.

[14] Lee S, Koppensteiner R, Kopp CW (2019). α-Hydroxybutyrate dehydrogenase is associated with atherothrombotic events following infrainguinal angioplasty and stenting. Sci Rep.

[15] Yu HT, Han HT, Li JJ (2019). Alpha-hydroxybutyrate dehydrogenase as a biomarker for predicting systemic lupus erythematosus with liver injury. Int Immunopharmacol.

[16] Li SK, Zhang YS, Li MJ (2017). Serum albumin, a good indicator of persistent organ failure in acute pancreatitis. BMC Gastroenterol.

[17] Banks PA, Bollen TL, Dervenis C (2013). Classification of acute pancreatitis-2012: revision of the Atlanta classification and definitions by international consensus. Gut.

[18] Mofidi R, Duff MD, Wigmore SJ (2006). Association between early systemic inflammatory response, severity of multiorgan dysfunction and death in acute pancreatitis. Br J Surg.

[19] Garg PK, Singh VP (2019). Organ failure due to systemic injury in acute pancreatitis. Gastroenterology.

[20] Yin X, Xu J, Zhang Q (2020). Quantification analysis of lactate dehydrogenase and C-reactive protein in evaluation of the severity and prognosis of the acute pancreatitis. Cell Mol Biol (Noisy-le-grand).

[21] Komolafe O, Pereira SP, Davidson BR (2017). Serum C-reactive protein, procalcitonin, and lactate dehydrogenase for the diagnosis of pancreatic necrosis. Cochrane Database Syst Rev.

[22] Yu HT, Han HT, Li JJ (2019). Alpha-hydroxybutyrate dehydrogenase as a biomarker for predicting systemic lupus erythematosus with liver injury. Int Immunopharmacol.

[23] Wojcicka J, Sienko J, Smolarczyk R (2005). Alpha-hydroxybutyrate dehydrogenase activity in intrahepatic cholestasis of pregnancy. Int J Gynaecol Obstet.

[24] Sun J, Su JW, Xie YR (2016). Plasma IL-6/IL-10 ratio and IL-8, LDH, and HBDH level predict the severity and the risk of death in AIDS patients with pneumocystis pneumonia. J Immunol Res.

[25] Doherty JR, Cleveland JL (2013). Targeting lactate metabolism for cancer therapeutics. J Clin Investig.

[26] Drent M, Cobben NA, Henderson RF (1996). Usefulness of lactate dehydrogenase and its isoenzymes as indicators of lung damage or inflammation. Eur Respir J.

[27] Zager RA, Johnson ACM, Becker K (2014). Renal cortical pyruvate depletion during AKI. J Am Soc Nephrol.

[28] Lozo VE, Miše K, Gudelj I (2019). Bronchoalveolar pH and inflammatory biomarkers in patients with acute exacerbation of chronic obstructive pulmonary disease. J Int Med Res.

[29] Ferriero R, Nusco E, De CR (2018). Pyruvate dehydrogenase complex and lactate dehydrogenase are targets for therapy of acute liver failure. J Hepatol.

[30] Fantin VR, St-Pierre J, Leder P (2006). Attenuation of LDH-A expression uncovers a link between glycolysis, mitochondrial physiology, and tumor maintenance. Cancer Cell.

